# Diffusible signal factor primes plant immunity against *Xanthomonas campestris* pv. *campestris* (*Xcc*) *via* JA signaling in *Arabidopsis* and *Brassica oleracea*

**DOI:** 10.3389/fcimb.2023.1203582

**Published:** 2023-06-19

**Authors:** Qian Zhao, Fang Liu, Cong Song, Tingting Zhai, Ziwei He, Limei Ma, Xuemeng Zhao, Zhenhua Jia, Shuishan Song

**Affiliations:** ^1^ Biology Institute, Hebei Academy of Sciences, Shijiazhuang, China; ^2^ Hebei Technology Innovation Center of Microbiological Control on Main Crop Disease, Shijiazhuang, China; ^3^ Shijiazhuang Academy of Agricultural and Forestry Sciences, Shijiazhuang, China

**Keywords:** quorum sensing, *Xanthomonas campestris* pv. *campestris*, plant immunity, priming, Jasmonic acid, DSF

## Abstract

**Background:**

Many Gram-negative bacteria use quorum sensing (QS) signal molecules to monitor their local population density and to coordinate their collective behaviors. The diffusible signal factor (DSF) family represents an intriguing type of QS signal to mediate intraspecies and interspecies communication. Recently, accumulating evidence demonstrates the role of DSF in mediating inter-kingdom communication between DSF-producing bacteria and plants. However, the regulatory mechanism of DSF during the *Xanthomonas*-plant interactions remain unclear.

**Methods:**

Plants were pretreated with different concentration of DSF and subsequent inoculated with pathogen *Xanthomonas campestris pv. campestris (Xcc)*. Pathogenicity, phynotypic analysis, transcriptome combined with metabolome analysis, genetic analysis and gene expression analysis were used to evaluate the priming effects of DSF on plant disease resistance.

**Results:**

We found that the low concentration of DSF could prime plant immunity against *Xcc* in both *Brassica oleracea* and *Arabidopsis thaliana*. Pretreatment with DSF and subsequent pathogen invasion triggered an augmented burst of ROS by DCFH-DA and DAB staining. CAT application could attenuate the level of ROS induced by DSF. The expression of *RBOHD* and *RBOHF* were up-regulated and the activities of antioxidases POD increased after DSF treatment followed by Xcc inoculation. Transcriptome combined with metabolome analysis showed that plant hormone jasmonic acid (JA) signaling involved in DSF-primed resistance to *Xcc* in Arabidopsis. The expression of JA synthesis genes (*AOC2, AOS, LOX2, OPR3* and *JAR1*), transportor gene (*JAT1*), regulator genes (*JAZ1* and *MYC2*) and responsive genes (*VSP2, PDF1.2* and *Thi2.1*) were up-regulated significantly by DSF upon Xcc challenge. The primed effects were not observed in JA relevant mutant *coi1-1* and *jar1-1*.

**Conclusion:**

These results indicated that DSF-primed resistance against *Xcc* was dependent on the JA pathway. Our findings advanced the understanding of QS signal-mediated communication and provide a new strategy for the control of black rot in *Brassica oleracea*.

## Introduction

Quorum sensing (QS) is a process of cell-to-cell communication depending on the population density of bacteria. By monitoring the concentration of QS signal molecules, bacteria regulate gene expression to coordinate their collective behaviors in order to improve their adaptability to complex nutrition and environmental conditions. The diffusible signal factor (DSF) family represents an intriguing type of QS signal found in diverse Gram-negative bacteria and mediates intraspecies and interspecies communication (He et al., 2023). DSF- mediated cell-to-cell signaling plays an important role in the virulence of the *Xanthomonas* group (Kakkar et al., 2015). In *Xanthomonas campestris* pv. *campestris* (*Xcc*), a global-threat phytopathogen that causes black rot on crucifers (Meyer et al., 2005), DSF regulates the production of several secreted virulence factors such as the exopolysaccharide (EPS) xanthan, extracellular cell wall-hydrolyzing enzymes, and glucan (Barber et al., 1997). Recently, increasing evidence shows that DSF mediates inter-kingdom communication between DSF-producing bacteria and their eukaryotic hosts (Kakkar et al., 2015; Li et al., 2019; Tran et al., 2020; Ma et al., 2021; Song et al., 2022; Zhai et al., 2022; Zhu et al., 2022; He et al., 2023). Kakkar et al. (2015) reported first that plants have evolved to recognize DSF signals and induce innate immunity against pathogen infection in a dose-dependent manner. Infiltration of leaves with 100 μM of DSF induced hypersensitive reaction (HR) -like symptom, callose deposition, programmed cell death, the accumulation of autofluorescent compounds and hydrogen peroxide (H_2_O_2_), and the expression of pathogenesis-related protein 1 (*PR-1*) gene and then reduced disease severity and *Xcc* growth in *Nicotiana benthamiana*, *Arabidopsis*, and rice (Kakkar et al., 2015). Intriguingly, infiltration of 10 μM of DSF did not induce a significant amount of callose deposition, while pre-infiltration with DSF and subsequent challenge with flg22 caused a substantial amount of callose deposition. This suggests that the application of a lower concentration of DSF may prime plants and influence their subsequent defense response (Kakkar et al., 2015).

The DSF modulation of plant immunity is highly spatiotemporally regulated and influences a diverse array of host biological systems (He et al., 2023). Tran et al. (2020) examined DSF effects on plant growth and PAMP-triggered immunity (PTI) responses. It was found that a low concentration of DSF with 5–10 μM promoted the primary root growth, while ≥ 25 μM of DSF inhibited root elongation in *Arabidopsis*. It is consistent with the report of Zhai et al. (2022). The promotion of the primary root elongation by 2 μM of DSF was due to the enhancement of cell division in the meristem zone and cell expansion in the elongation zone and involvement with auxin signaling (Zhai et al., 2022).

DSF measuring 25 μM impaired flg22-elicited stomatal closure, callose deposition, and reactive oxygen species (ROS) burst. Such dampening of PTI responses was a consequence of a DSF-induced phytosterol production that impaired both clathrin-mediated endocytosis (CME) for internalizing FLS2 and FLS2 nano-clustering on the cell surface, which therefore desensitized *Arabidopsis* immune responses to the bacterial flagellum (Tran et al., 2020). A significant reduction of actin assembly was observed after DSF treatment in a dose-dependent manner starting from 25 μM (Ma et al., 2021). DSF elevated *Arabidopsis* cellulose production, which caused fewer restraints on lateral diffusion and nano-clustering of CW-bound type-1 formin, and thus further led to less actin nucleation during plant–bacteria communication (Ma et al., 2021). These pieces of evidence show that DSF-mediated plant–bacteria interaction is much more complex. The regulatory mechanism of DSF during the *Xanthomonas*–plant interactions remains unclear.

Plant hormones play major roles in the establishment of signaling networks to regulate plant growth and stress-related responses (Ruan et al., 2019). Jasmonic acid (JA)- and salicylic acid (SA)-mediated signaling pathways are mainly related to plant resistance, prompting plant responses to external damage (mechanical, herbivore, and insect damage) and pathogen infection (Ruan et al., 2019). Evidence demonstrates that JA signaling and SA signaling are involved in QS molecule-primed plant immunity. *N*-3-Oxo-tetradecanoyl-l-homoserine lactone (3OC14-HSL), one of the QS molecules, was reported to prime plants for cell wall reinforcement and induce resistance to bacterial pathogens *via* the SA/oxylipin pathway (Schenk et al., 2014). Another QS molecule, *N*-3-oxo-octanoyl-l-homoserine lactone (3OC8-HSL), primes *Arabidopsis* defense response to hemi-biotrophic bacterial infection depending on the SA signaling pathway (Liu et al., 2020), while it primes *Arabidopsis* resistance against necrotrophic pathogen by coordinating JA and auxin signaling pathways (Liu et al., 2022). Different from a single *N*-acyl homoserine lactone (AHL) molecule, the combination of AHL molecules including 3OC14-HSL, *N*-3-oxo-dodecanoyl-l-homoserine lactone (3OC10-HSL), 3OC8-HSL, and *N*-3-oxo-hexanoyl-l-homoserine lactone (3OC6-HSL) primes *Arabidopsis* defenses *via* JA metabolism (Duan et al., 2023). A recent study demonstrated that plant SA signal directly acts on the QS system of the invading pathogen *Xcc* to affect its virulence by inducing the turnover of the DSF family QS signal *via* a pH-dependent manner (Song et al., 2022). However, the role of plant hormones in DSF-mediated *Xanthomonas*–plant interactions and the associated underlying molecular mechanisms are not yet fully understood.

In this study, we examined the effects of DSF on triggering the plant defense responses with a low concentration (0.1–10 μM), which was considered as the priming concentration during plant-*Xcc* interaction (Kakkar et al., 2015). It was found that 2 μM of DSF could prime plant immunity effectively against *Xcc* in both *Brassica oleracea* and *Arabidopsis thaliana*. DSF pretreatment decreased the disease symptoms and *Xcc* proliferation on plant leaves. ROS burst, H_2_O_2_ accumulation, and antioxidase activation were observed after DSF treatment followed by *Xcc* inoculation. Transcriptome combined with metabolome analysis showed that plant hormone JA signaling was involved in DSF-primed resistance to *Xcc* in *Arabidopsis*. Upregulating gene expression and desensitizing *Arabidopsis* mutants of JA signaling confirmed that DSF-primed resistance against *Xcc* was dependent on the JA pathway. Our findings advanced the understanding of QS signal-mediated communication and provide a new strategy for the control of black rot in *B. oleracea*.

## Materials and methods

### Plant materials and growth conditions

*A. thaliana* (L.) cv. Columbia-0 and the homozygous T-DNA inserted mutants *coi1-1* (CS4144) and *jar1-1* (CS8072) were obtained from The *Arabidopsis* Information Resource (TAIR http://www.arabidopsis.org). *Arabidopsis* seeds were surface-sterilized by 75% (v/v) ethanol and 20% (v/v) NaClO and geminated on Murashige and Skoog (MS) Polygel medium (Murashige and Skoog, 1962) adjusted to pH 5.8. The seeds were stratified at 4°C for 2 days and then grown in a growth chamber at 22°C ± 2°C and a 16- h light/8- h dark cycle with a light intensity of 120 μmol·m^2^·s^−1^. Ten- day-old seedlings grown vertically on MS polygel plates were transplanted into a floating hydroponic system containing sterile Hoagland medium for another 10 days. These seedlings were used for DSF or pathogenic treatments. Fertilized substrate TS1 (Klasmann-Deilmann GmbH, Saterland, Germany) and vermiculite (1:2) were used for soil culture.

Cabbage (*B. oleracea* var. *capitata* L.) seeds were soaked in water until germination and then transplanted into a floating hydroponic system containing sterile MS medium at 25°C ± 2°C and under 16/8-h photoperiod (light/dark) with a light intensity of 120 μmol·m^2^·s^−1^. Fertilized substrate TS1 (Klasmann-Deilmann GmbH, Germany) and vermiculite (1:1) were used for soil culture.

### Bacterial strains and growth conditions

*X. campestris* pv. *campestris* (*Xcc*) wild-type strain 8004 and its *rpfF* gene deletion mutant strain Δ*rpfF* were grown in NYG agar or NYG medium containing 25 µg/ml of rifampicin at 28°C. *Xcc* was cultured overnight with shaking at 200 rpm, then collected by centrifugation, washed, and re-suspended with 10 mM of sterile MgSO_4_. Bacterial growth was measured in a spectrophotometer at 600 nm. The bacterial inoculum cells were diluted to a final density of 1 × 10^8^ CFU/ml by 10 mM of sterile MgSO_4_.

### DSF treatment

DSF (*cis*-11-methyl-2-dodecenoic acid) was purchased from Sigma-Aldrich (St. Louis, MO, USA), stored dry, diluted as 10- mM stock solutions in dH_2_O, and sterilized by passing them through a 0.22-µm filter just prior to use. Indicated concentrations of DSF were used as the treatments in our experiments. The untreated plants were taken as the control.

### Pathogenicity tests

To inoculate *Arabidopsis* with *Xcc*, the roots of 20-day-old hydroponic seedlings were pretreated with different concentrations (0 nM, 100 nM, 1 µM, 2 µM, 5 µM, and 10 µM) of DSF for 48 h; then, the leaves were sprayed with 1 × 10^8^ CFU/ml of *Xcc*. After inoculation for 24, 48, and 72 h, 100 mg of leaf tissues was harvested and homogenized in 10 mM of MgSO_4_. Then, the homogenate was diluted and plated onto YNG agar medium containing 25 µg/ml of rifampicin for colony- forming unit (CFU) counting. Three independent biological experiments were conducted with three technical replications each. Detached leaves from 4-week-old soil-cultured *Arabidopsis* seedlings were soaked in sterile dH_2_O or 2 µM of DSF for 48 h. Then, leaves were placed in sterile Petri dishes containing two layers of moist filter paper to maintain high humidity. Leaves were inoculated with 1 × 10^8^ CFU/ml of *Xcc* and cultured at 28°C. The disease symptoms were investigated at 72 h after inoculation. Sterile dH_2_O treatments were taken as the control. Three independent biological experiments were conducted, and each of them included at least 18 leaves.

Cabbage soil-cultured in four leaves and one shoot period was used for treatments. Two pathogenic inoculation methods were used for cabbage. The suspension inoculation method was performed by making a small cross at the petiole on the back of cabbage leaves and dropping 10 µl of bacterial suspension with the cut exposed to air until it is absorbed. The leaf cutting inoculation method was performed by cutting a wound perpendicular to the main vein of the leaf with scissors dipped in the bacterial suspension. Detached leaves were soaked in sterile dH_2_O or 2 µM of DSF for 48 h, placed in sterile Petri dishes containing two layers of moist filter paper, suspension- inoculated with 1 × 10^8^ CFU/ml of *Xcc*, and cultured at 28°C. CFU counting was conducted at 72 h, and the disease symptom was investigated 9 days after inoculation. In the pot experiment, the roots of cabbage were treated with sterile dH_2_O or 2 µM of DSF for 48 h when the soil was almost dry, and the leaves of cabbage underwent suspension inoculation or leaf cutting inoculation with 1 × 10^8^ CFU/ml of *Xcc* and cultured at 28°C. The disease symptom was investigated 21 days after inoculation. Sterile dH_2_O treatments were taken as the control. Three or six independent biological experiments were conducted, and each of them included at least eight leaves and six seedlings.

### DCFH-DA staining

*Arabidopsis* seeds were surface-sterilized and geminated on MS Polygel medium for 3–5 days. The seedlings were transplanted to 1/2 MS Polygel medium containing 2 µM of DSF when the root length was up to 1.5–2 cm. DSF-untreated seedlings were taken as the control. After 1, 3, 6, 12, 24, 36, and 48 h of treatments, the roots were stained by 10 µM of 2′,7′-dichlorofluorescin diacetate (DCFH-DA; Sigma-Aldrich, Taufkirchen, Germany) for 1 h and washed three times by 1/2 MS medium, and then the green fluorescence intensity was examined by a laser scanning confocal microscope (excitation, 488 nm; emission, 500-550 nm; Leica SP8, Leica Biosystems, Wetzlar, Germany).

To determine H_2_O_2_ accumulation induced by DSF, 50 µg/ml of catalase (CAT) was used with DSF treatment. DCFH-DA staining was performed, and fluorescence intensity was scanned after 24 h of treatment. Three independent experiments were performed with 8–19 roots for each treatment.

### DAB staining

Detached leaves of 4-week- old *Arabidopsis* seedlings were pretreated with 2 µM of DSF for 2 days and then inoculated with 1 × 10^8^ CFU/ml of *Xcc* for 0, 3, 6, 12, 24, and 48 h. Leaves were soaked in 1 mg/ml of 3,3′-diaminobenzidine (DAB; Sigma-Aldrich, Germany) and shaken at 80–100 rpm for 4–8 h at 28°C in the dark. Then, 95% ethanol was used to bleach the leaves. DSF-untreated leaves were taken as the control. The experiments were performed three times with six leaves for each treatment.

### Hydrogen peroxide determination

Twenty-day- old hydroponic *Arabidopsis* seedlings were pretreated with or without 2 μM of DSF in roots for 48 h and then spray-inoculated with 10^8^ CFU/ml of pathogen *Xcc*. Leaves of plants were harvested at 48 h after DSF pretreatment and 48 h after *Xcc* inoculation. H_2_O_2_ content was determined using a Hydrogen Peroxide assay kit according to the manufacturer’s instructions (Solarbio Science & Technology Co., Ltd., Beijing, China). H_2_O_2_ reacts with titanium sulfate to form a yellow titanium peroxide complex, which has characteristic absorption at 665 nm. Three independent biological experiments were conducted with three technical replicates each.

### Peroxidase activity determination

Twenty-day- old hydroponic *Arabidopsis* seedlings were pretreated with or without 2 μM of DSF in roots for 48 h and then spray-inoculated with 10^8^ CFU/ml of pathogen *Xcc*. Leaves of plants were harvested at 48 h after DSF pretreatment and 48 h after *Xcc* inoculation. The enzymatic activity of peroxidase (POD) was determined using POD Activity Assay Kit according to the manufacturer’s instructions (Solarbio Science & Technology Co., Ltd., Beijing, China). Three independent biological experiments were conducted with three technical replicates each.

### RNA-seq

Twenty-day- old hydroponic *Arabidopsis* seedlings were pretreated with or without 2 μM of DSF in roots for 48 h and then spray-inoculated with 10^8^ CFU/ml of pathogen *Xcc*. Leaves of plants were harvested at 48 h after DSF pretreatment and 48 h after *Xcc* inoculation. Total RNA was extracted from treated and control plants using TRIzol^®^ Reagent according to the manufacturer’s instructions (Magen). Paired-end libraries were prepared using ABclonal mRNA-seq Lib Prep Kit (ABclonal, Wuhan, China) following the manufacturer’s instructions. The library preparations were sequenced on an Illumina Novaseq 6000, and 150 bp paired-end reads were generated. Raw data of fastq format were first processed through in-house perl scripts by r emoving the adapter sequence and filtering out low- quality and N ratio greater than 5% reads to obtain clean reads that can be used for subsequent analysis. Then, clean reads were separately aligned to the reference genome with orientation mode using HISAT2 software (http://daehwankimlab.github.io/hisat2/) to obtain mapped reads. FeatureCounts (http://subread.sourceforge.net/) were used to count the read numbers mapped to each gene. Then, the Fragments per Kilobase Million (FRKM) of each gene was calculated based on the length of the gene and the reads count mapped to this gene. Differential expression analysis was performed using the DESeq2 (http://bioconductor.org/packages/release/bioc/html/DESeq2.html), DEGs with | log2FC | > 1 and p adjust < 0.05 were considered to be significantly differentially expressed genes. ClusterProfiler R software package was used for Gene Ontology (GO) function enrichment and Kyoto Encyclopedia of Genes and Genomes (KEGG) pathway enrichment analyses. When p < 0.05, it is considered that the GO or KEGG function is significantly enriched. The data discussed in this study have been deposited in NCBI’s Gene Expression Omnibus and are accessible through GEO Series accession number GSE229511 (https://www.ncbi.nlm.nih.gov/geo/query/acc.cgi?acc=GSE229511).

### Targeted metabolomics analysis and phytohormone determination

Twenty-day- old hydroponic *Arabidopsis* seedlings were pretreated with or without 2 μM of DSF in roots for 48 h and then spray-inoculated with 10^8^ CFU/ml of pathogen *Xcc*. Leaves of plants were harvested at 48 h after DSF pretreatment and 48 h after *Xcc* inoculation. Targeted metabolomics analysis based on multiple reaction monitoring (MRM) technology was performed. Samples were ground using liquid nitrogen, and metabolites were extracted by acetonitrile solution (1%FA). Samples were separated by Agilent 1290 Infinity LC ultra-high- performance liquid chromatography (Agilent, Santa Clara, CA, USA). The conditions of ultra-high-performance liquid chromatography (UHPLC) are as follows: the mobile phase system is 1%FA solution (A) and acetonitrile (B); the chromatographic column is ACQUITY UPLC BEH C18 1.7 μm, 2.1 mm × 100 mm column (Waters, Milford, MA, USA); column temperature, 40°C; liquid velocity, 0.4 ml/min. Gradient eluted program: 0–20 min, 0%–90%B; 20–25 min, 90%B; 25–27 min, 90%–0%B; 27–30 min, 0%B. Mass spectrometric analysis was performed by Thermo Scientific TSQ Quantiva (Thermo, Waltham, MA, USA). The conditions were as follows: sheath gas flow, 30 Arb; auxiliary gas flow, 10 Arb; sweep gas flow, 8 Arb; collision gas, 1.5 mTorr; ion transfer tube temperature, 350°C; vaporizer temperature, 300°C; spray voltage, 3.0 kV. Thermo Scientific Xcalibur 2.1 software was used for data analysis. The Quantitative results of phytohormones were calculated based on the standard curve.

Samples were harvested at 0, 6, 12, 24, and 48 h after *Xcc* inoculation in DSF 48 h-pretreated seedlings or non-DSF-pretreated seedlings (taken as the control). The contents of JA and JA-Ile were determined according to the above method.

### qRT-PCR

Twenty-day- old hydroponic cultured *Arabidopsis* and cabbage seedlings were treated with 2 µM of DSF for 0, 6, 12, 24, and 48 h and then harvested for RNA extraction. Untreated plants were used as controls. Other treatments were that DSF- pretreated seedlings for 48 h were inoculated with 1 × 10^8^ CFU/ml of *Xcc* for 0, 6, 12, 24, and 48 h and then harvested for RNA extraction. DSF-untreated plants were used as controls. Total RNA was extracted using the RNAiso Plus reagent (TaKaRa, Dalian, China). cDNA was synthesized using the PrimeScript^®^ RT Reagent Kit with gDNA Eraser (TaKaRa, Dalian, China) according to the manufacturer’s instructions. SYBR Premix Ex Taq was purchased from TaKaRa (Shiga, Japan). For the relative quantification of gene expression, the comparative CT method (Livak and Schmittgen, 2001) was used with the 7500 Real Time PCR System (Applied Biosystems, Foster City, CA, USA). The amount of target was normalized to the endogenous reference gene *ACTIN2/8* (AT3G18780) in *Arabidopsis* and *BoACTIN1* (AF044573) in cabbage. For technical control, each sample was repeated three times on the same 96-well plate. Each data point represents the average of three independent experiments. A 1.5-fold increase (ratio > 1.5) or 1.5-fold decrease (ratio < 0.8) in expression in the treated plants compared with the untreated plants (the control) was considered as upregulation or downregulation, respectively, in relation to DSF response. The specific primers used for qRT-PCR are shown in **Supplementary Table 1**.

### Statistical analysis

For all experiments, the overall data were statistically analyzed in the DPS v7.05 program. Univariate and multivariate analyses (ANOVA) with Duncan’s new multiple- range tests (p < 0.05) were used. All data were represented as means ± SD of three or six independent experiments.

## Results

### DSF protects *Arabidopsis* from *Xcc* infection

**Figure 1:**
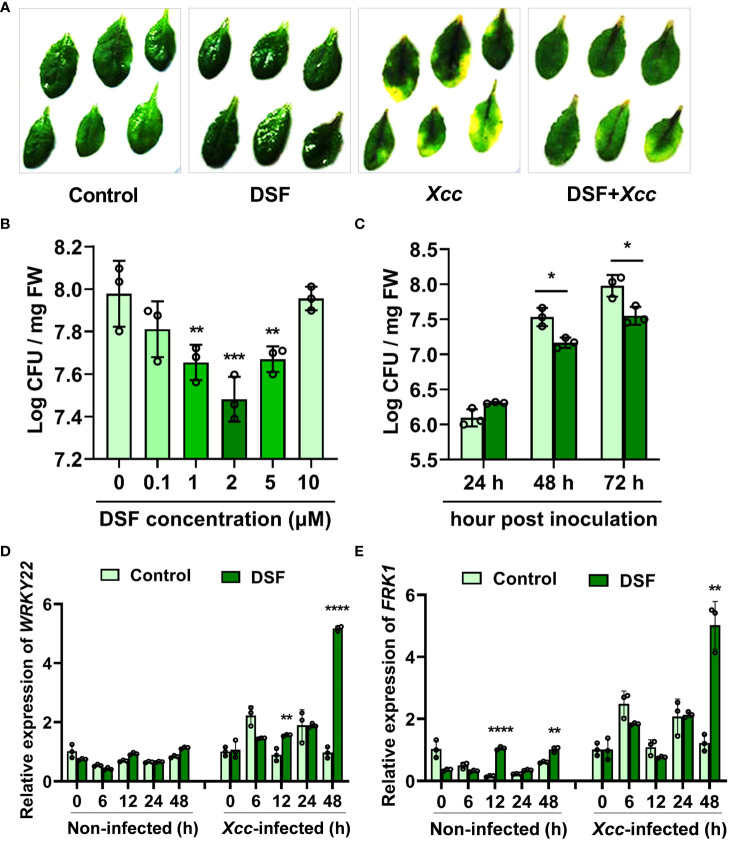
Enhanced resistance of DSF-treated *Arabidopsis* against *Xcc*. **(A)** Symptoms of *Xcc* infection on wild-type *Arabidopsis* Col-0 pretreated with 2 µM of DSF. The disease symptoms were recorded at 72 h after inoculation. **(B)** Priming effects of different concentrations of DSF on *Xcc* growth in *Arabidopsis*. The roots of hydroponically grown plants were pretreated with indicated concentrations of DSF for 48 h prior to foliar inoculation with 10^8^ CFU/ml of *Xcc*. CFUs were counted at 72 hpi. **(C)** Inhibitory effect of DSF on *Xcc* growth in *Arabidopsis*. The seedlings were pretreated with 2 µM of DSF for 48 h prior to inoculation with 10^8^ CFU/ml of *Xcc*. CFUs were counted at indicated hpi. **(D)** The expression level of PTI marker gene *WRKY22*. **(E)** The expression level of PTI marker *FRK1*. The seedlings were pretreated with 2 µM of DSF for 48 h prior to inoculation with 10^8^ CFU/ml of *Xcc*. Samples were harvested at indicated time points for qPCR. Three independent experiments were performed with three replicates in each experiment, and similar results were obtained. Values are means ± SD of three independent experiments. Asterisks indicate statistically significant differences (ANOVA test, *p < 0.05, **p < 0.01, ***p < 0.001, ****p < 0.0001). DSF, diffusible signal factor; CFU, colony-forming unit; hpi, post-inoculation.

The influence of bacterial QS molecule DSF on priming plant defense responses was analyzed. Detached leaves from 3–4-week- old soil-cultured *Arabidopsis* were pretreated with 2 µM of DSF for 48 h prior to spray inoculation with 10^8^ CFU/ml of pathogen *Xcc*. The disease symptoms were observed at 72 h after inoculation. The leaves pretreated with DSF exhibited no significant *Xcc* symptoms, while the un-pretreated leaves turned yellow (**Figure 1A**). To evaluate the dose-dependent induction effects of DSF on *Xcc*, a series concentration of DSF, ranging from 0.1 to 10 µM, were used to pretreat the roots of hydroponic *Arabidopsis* seedlings for 48 h and then spray-inoculated with *Xcc* in leaves. The bacterial CFUs in the leaf tissue were counted at 72 h post- inoculation (hpi). Pathogen proliferation was significantly inhibited by 1, 2, and 5 µM of DSF (**Figure 1B**). The optimum concentration of DSF treatment that was screened was 2 µM. To monitor the disease progression on the leaves of DSF-pretreated plants, CFUs were monitored for 72 h after pathogen infection. While bacterial titer gradually increased in the leaves of *Arabidopsis* without DSF pretreatment, pathogen proliferation was significantly reduced in the DSF-pretreated plants after 48- and 72- h inoculation (**Figure 1C**). The expression of PTI marker genes *WRKY22* and *FRK1* was detected by qPCR in seedlings pretreated by DSF prior to *Xcc* infection. DSF induced the expression of *WRKY22* and *FRK1* to dramatically increase at 48 hpi, and the fold change was up to 5.33 and 4.1, respectively (**Figures 1D, E**). These results indicated that DSF may play an important role in protecting plants from *Xcc* infection.

In consideration of DSF being the main QS molecule generated by *Xcc*, the *rpfF* gene deletion mutant strain Δ*rpfF*, which is not able to produce DSF, was introduced to detect the effect of DSF on priming plant resistance to pathogens. First, exogenous DSF had no influence on bacterial growth in both *Xcc* wild-type strain 8004 and *Xcc*Δ*rpfF* (**Supplementary Figure 1B**). Pathogen proliferation of *Xcc*Δ*rpfF* was also inhibited by exogenous DSF application, which has no difference compared to *Xcc*8004, in *Arabidopsis* (**Supplementary Figure 1A**). It indicated that the DSF priming effect was not influenced by endogenous DSF generated by *Xcc*.

### DSF elicits ROS burst in *Arabidopsis* roots

**Figure 2:**
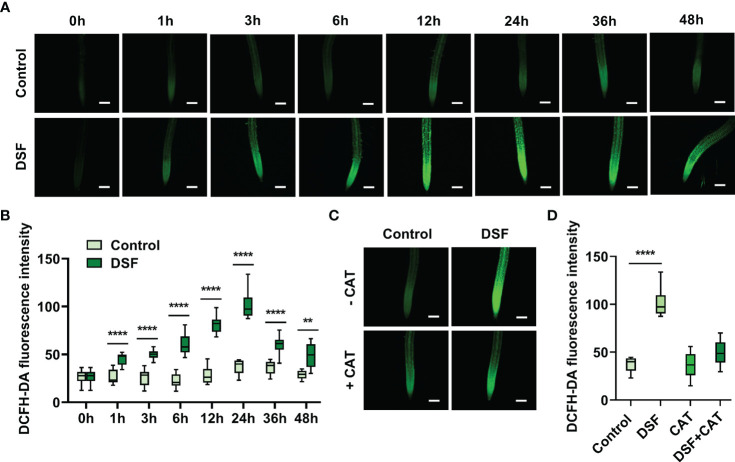
Effects of DSF on ROS burst by DCFH-DA staining in *Arabidopsis* root. **(A)** Green fluorescence of primary roots in 5-day-old *Arabidopsis* Col-0 seedlings exposed to 2 µM of DSF for indicated time. Scale bar = 100 μm. **(B)** Intensity of green fluorescence from panel **(A, C)** CAT effect on fluorescence diminishing; 50 μg/ml of CAT and 2 µM of DSF co-treated the roots of 5-day-old *Arabidopsis* seedlings for 24 h, and green fluorescence was observed. Scale bar = 100 μm. **(D)** Intensity of green fluorescence from panel **(C)** Three independent experiments were performed and 8–19 roots each treatment. Values are means ± SD of three independent experiments. Asterisks indicate statistically significant differences (ANOVA test, **p < 0.01, ****p < 0.0001). DSF, diffusible signal factor; ROS, reactive oxygen species; CAT, catalase.

DCFH-DA probe was used to monitor the oxidation–reduction process in cells and detect intracellular ROS production, especially H_2_O_2_, with the intensity of green fluorescence (Lyublinskaya et al., 2017). The roots of *Arabidopsis* treated with DSF for 0, 1, 3, 6, 12, 24, 36, and 48 h were stained by DCFH-DA. An increasing enhancement of green fluorescence was observed along with DSF treatment time extension, while there were no significant changes in the untreated control (**Figure 2A**). The peak of the intensity of green fluorescence was at 24 h after DSF treatment (**Figure 2B**). To test H_2_O_2_ accumulation induced by DSF, CAT, one of the main enzymes that catalyze the breakdown of hydrogen peroxide, was applied with DSF treatment for 24 h in DCFH-DA staining assay. CAT significantly reduced the intensity of green fluorescence induced by DSF (**Figures 2C, D**). These results suggested that DSF can elicit ROS burst and H_2_O_2_ accumulation in *Arabidopsis*.

### DSF triggers H_2_O_2_ accumulation upon pathogen infection

**Figure 3:**
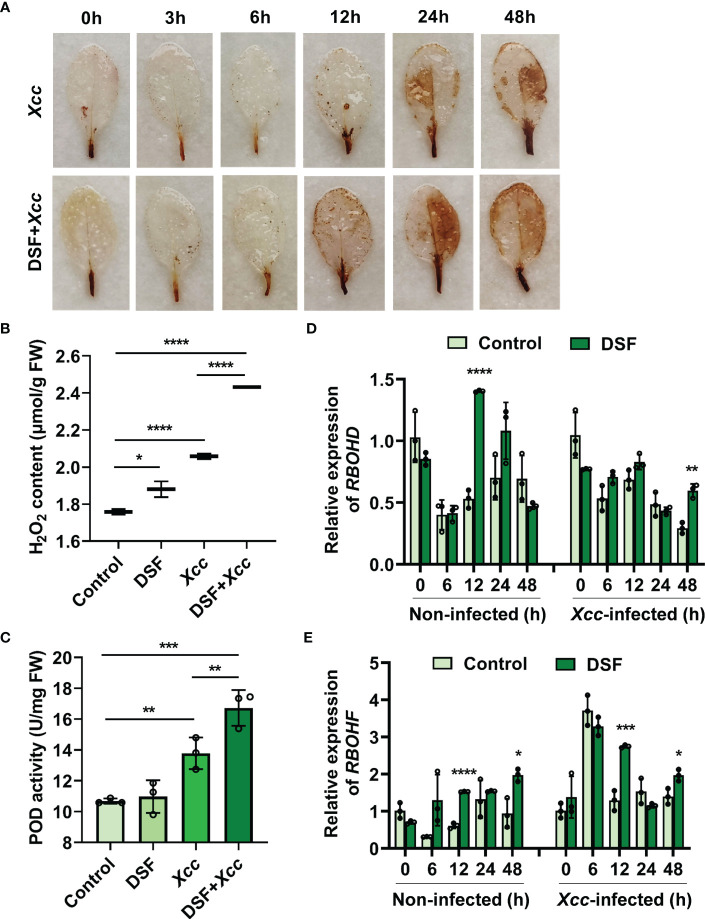
Effects of priming by DSF and pathogen challenge on H_2_O_2_ accumulation. **(A)** H_2_O_2_ accumulation in leaves stained by diaminobenzidine (DAB). Detached leaves of *Arabidopsis* pretreated with or without 2 µM of DSF for 48 h and then inoculated with 10^8^ CFU/ml of *Xcc* inoculation. Leaves at indicated hpi were stained by DAB for 24 h. After de-staining, the leaves were photographed. The experiments were performed with six leaves per treatment, and similar results were obtained in three independent experiments. **(B)** H_2_O_2_ content in *Arabidopsis* leaves. **(C)** POD activity in *Arabidopsis* leaves. Control presents the sample with distilled water treatment for 48 h. DSF presents DSF treatment for 48 h. *Xcc* presents *Xcc* inoculation for 48 h. DSF+*Xcc* presents DSF pretreatment for 48 h followed by *Xcc* inoculation for another 48 h. Control is the untreated seedlings that grew synchronously with DSF- treated seedlings. **(D)** The expression level of *RBOHD*. **(E)** The expression level of *RBOHF*. The seedlings were pretreated with 2 µM of DSF for 48 h prior to inoculation with 10^8^ CFU/ml of *Xcc*. Samples were harvested at indicated time points for qPCR. Values are means ± SD of three independent experiments. Asterisks indicate statistically significant differences (ANOVA test, *p < 0.05, **p < 0.01, ***p < 0.001, ****p < 0.0001). DSF, diffusible signal factor; CFU, colony-forming unit; POD, peroxidase; hpi, post-inoculation.

H_2_O_2_ accumulation in leaves was analyzed first by DAB staining. Detached leaves of *Arabidopsis* pretreated with 2 µM of DSF were stained after *Xcc* inoculation at different time points. Brown color in DSF-pretreated leaves was deeper than in un-pretreated leaves after 12 hpi, and the most obvious difference appeared at 24 hpi (**Figure 3A**). Then, the level of H_2_O_2_ in leaves was determined by titanium sulfate colorimetry at 24 h post *Xcc* inoculation. A very strong accumulation of H_2_O_2_ was detected in DSF-pretreated leaves after pathogen infection (**Figure 3B**). Meanwhile, the activity of POD, one of the main H_2_O_2_ scavenging enzymes, also rose rapidly when induced by DSF pretreatment with subsequent pathogen invasion (**Figure 3C**). The gene expression of respiratory burst oxidase homolog proteins D and F (*RBOHD* and *RBOHF*) were detected by qPCR in seedlings pretreated by DSF prior to *Xcc* infection. RBOHD and RBOHF were the calcium- dependent NADPH oxidases that play important roles in generating ROS in plant immunity. DSF induced the expression of *RBOHD* and *RBOHF* to increase at 12 h after DSF treatment and at 12 and 48 h after *Xcc* infection (**Figures 3D, E**). The expression of RBOHF was greatly triggered at 6 h after *Xcc* infection, while there was no significant difference between DSF pretreatment and the un-pretreated control perhaps due to the *Xcc* challenge. These results indicated that DSF pretreatment could trigger RBOH-mediated ROS production when the plant underwent the *Xcc* challenge.

### Transcriptional changes primed by DSF in *Arabidopsis*

To clarify the mechanism of DSF priming the plant immunity against *Xcc*, an RNA sequencing approach was employed to analyze the changes in transcriptomic gene expression in *Arabidopsis*. The seedlings were pretreated by DSF for 48 h following *Xcc* infection for another 48 h. DSF- untreated seedlings with synchronous growth were taken as blank control, and *Xcc* -inoculated seedlings without DSF pretreatment were taken as pathogen control. A total of 230 differentially expressed genes (DEGs) including 191 upregulated genes and 39 downregulated genes were identified in DSF- treated seedlings compared to untreated control. There were a total of 227 DEGs including 205 genes upregulated and 22 genes downregulated in DSF- pretreated seedlings prior to the *Xcc* challenge compared to *Xcc* -inoculated seedlings without DSF pretreatment. A total of 82 upregulated DEGs were found in both DSF-treated seedlings and DSF-pretreated seedlings with *Xcc* infection. There were no common downregulated DEGs in DSF-treated seedlings and DSF-pretreated seedlings following *Xcc* invasion (**Supplementary Figure 2**).

To verify the data of RNA-seq, qPCR was used to analyze the gene expression levels of 10 selected DEGs involved in JA signaling, Ca^2+^ signaling, MAPK signaling, ethylene signaling, auxin signaling, and leucine-rich repeat receptor-like protein kinases. Similar results of up- or down regulation and the fold changes of gene expression were obtained by the qPCR method, which confirmed the reliability of RNA-seq results (**Supplementary Table 2**).

**Figure 4:**
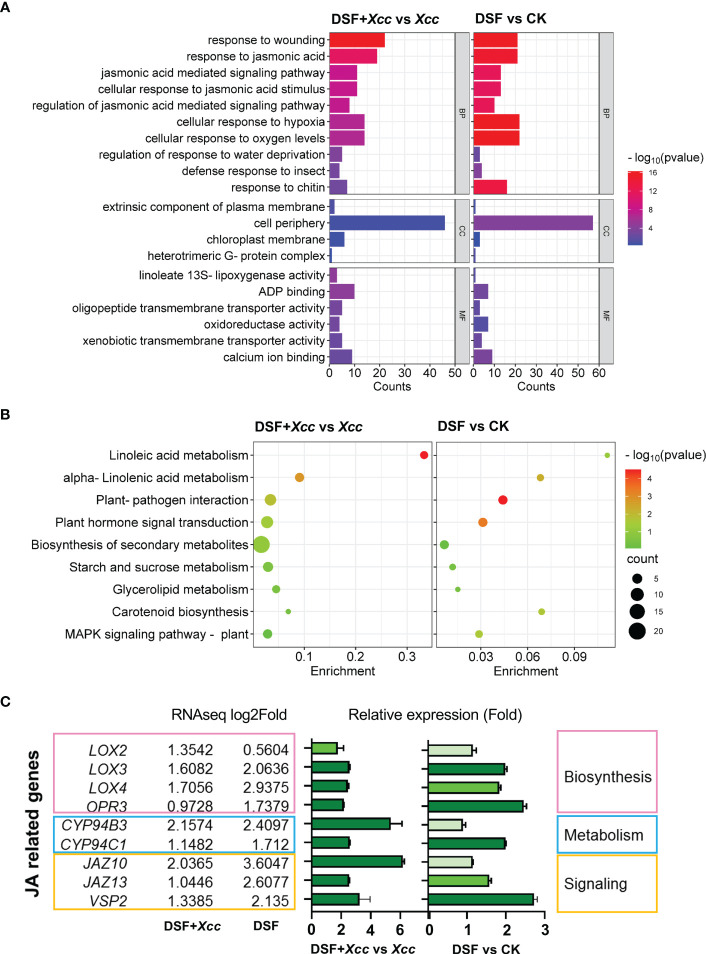
Transcriptional changes primed by DSF in *Arabidopsis*. The seedlings were pretreated with 2 µM of DSF for 48 h prior to inoculation with 10^8^ CFU/ml of *Xcc*. RNA sequencing was performed. Differentially expressed genes (DEGs) were analyzed between DSF- treated seedlings and untreated control seedlings (DSF *vs.* CK) or DSF- pretreated seedlings followed by *Xcc* infection and *Xcc* -inoculated seedlings without DSF pretreatment (DSF+*Xcc vs. Xcc*). The DEGs were filtered by threshold (p adjust < 0.05, log2foldchange > 1) with three independent replicates. **(A)** GO enrichment (top 20 terms). **(B)** KEGG pathway enrichment (top 9 terms). **(C)** The transcriptional level of JA relative genes upregulated in RNA-seq data and verified by qPCR. RNA-seq values are means of three replicate sample data, and qPCR values are means ± SD of three independent experiments. Light green indicates the qPCR ratio ≈ 1 (no change), medium green indicates 1.5 < qPCR ratio < 2 (slight upregulation), and dark green indicates qPCR ratio > 2 (significant upregulation). DSF, diffusible signal factor; CFU, colony-forming unit; GO, Gene Ontology; KEGG, Kyoto Encyclopedia of Genes and Genomes.

To reveal the biological function of DEGs primed by DSF, GO and KEGG pathway enrichment analyses were performed. The top 10 enrichment terms in Biological Process (BP) were response to wounding, response to JA, JA mediated signaling pathway, cellular response to JA stimulus, regulation of JA mediated signaling pathway, cellular response to hypoxia, to oxygen levels, regulation of response to water deprivation, defense response to insect, and response to chitin (**Figure 4A** and **Supplementary Tables 3**, **4**). In Cellular Component (CC) and Molecular Function (MF), the terms chloroplast membrane and linoleate-13*S*-lipoxygenase activity were enriched (**Figure 4A** and **Supplementary Tables 3**, **4**). Chloroplast is the main reaction site of JA biosynthesis, and linoleate-13*S*-lipoxygenase is the key enzyme to initiate JA biosynthesis (Ruan et al., 2019). These pieces of evidence suggested that JA signaling might be involved in the DSF priming effect.

The KEGG pathways that DSF primed include linoleic acid metabolism, alpha-linolenic acid metabolism, plant–pathogen interaction, plant hormone signal transduction, biosynthesis of secondary metabolites, and MAPK signaling pathway (**Figure 4B** and **Supplementary Tables 5**, **6**). Alpha-linolenic acid, one of the linoleic acids, is the substrate of LOX2 in the initiation of JA biosynthesis (Li et al., 2021). Alpha-linolenic acid metabolism pathway involved in DSF priming further indicated that JA signaling might be involved in the DSF priming effect.

Upregulated DEGs involved in JA biosynthesis, metabolism, and signaling were confirmed by qPCR. *LOX2*, *LOX3*, *LOX4*, and 12-oxophytodienoate reductase 3 (*OPR3*) belong to JA biosynthesis genes (Li et al., 2021). *LOX3*, *LOX4*, and *OPR3* were induced significantly by DSF treatment, while the expression of *LOX2*, *LOX3*, and *LOX4* was more increased induced by DSF after the *Xcc* challenge (**Figure 4C**). JA metabolism genes include *Cytochrome P450 94B3* (*CYP94B3*) and *CYP94C1*, which catalyzed JA-Ile into 12-OH-JA and 12-COOH-JA (Ruan et al., 2019). Enhanced expression of these two genes was observed in seedlings pretreated by DSF prior to *Xcc* infection (**Figure 4C**). JA signaling genes such as *JAZ10*, *JAZ13*, and *VSP2* were also induced greatly by DSF after *Xcc* infection (**Figure 4C**). These consistent results suggested that JA signaling may be involved in the DSF priming process.

In addition, hypoxia response, calcium ion binding, heterotrimeric G protein, MAPK signaling, and carotenoid biosynthesis (ABA biosynthesis relevance) were also enriched in GO and KEGG analyses (**Figures 4A, B**). It was suggested that DSF might play a role in these areas.

### Metabolomics analysis and phytohormone changes primed by DSF

**Figure 5:**
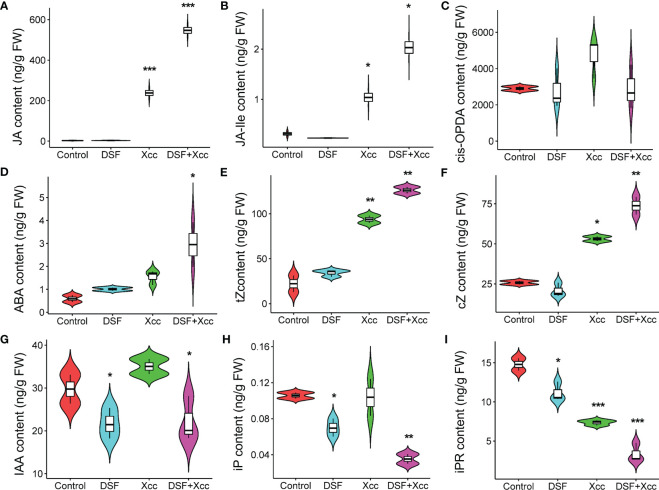
Metabolomics changes primed by DSF in *Arabidopsis*. The seedlings were pretreated with 2 µM of DSF for 48 h prior to inoculation with 10^8^ CFU/ml of *Xcc*. Phytohormones were extracted to test the level changes between DSF treatment and untreated control (48 h) or between DSF pretreatment and un-pretreatment prior to *Xcc* infection (48 hpi). **(A)** JA, **(B)** JA-Ile, **(C)** *cis*-OPDA, **(D)** ABA, **(E)** tZ, **(F)** cZ, **(G)** IAA, **(H)** iP, and **(I)** iPR. Values are means of three duplicate sample data. Asterisks indicate statistically significant differences (ANOVA test, *p < 0.05, **p < 0.01, ***p < 0.001). DSF, diffusible signal factor; CFU, colony-forming unit; hpi, post-inoculation.

To further investigate the mechanism of DSF priming the plant immunity against pathogen *Xcc*, metabolomics analysis was used, and the level of phytohormones was determined in *Arabidopsis*. The seedlings were pretreated by DSF for 48 h following *Xcc* infection for another 48 h. DSF- untreated seedlings with synchronous growth were taken as blank control, and *Xcc* -inoculated seedlings without DSF pretreatment were taken as pathogen control. The contents of 13 phytohormones were determined including SA, abscisic acid (ABA), 1-aminocyclopropane-1-carboxylic acid (ACC), indoleacetic acid (IAA), JA, JA-isoleucine conjugate (JA-Ile), 12-oxo-phytodienoic acid (*cis*-OPDA), *N* ^6^-dimethylallyladenine (iP), *N* ^6^-dimethylallyladenine riboside (iPR), trans-zeatin (tZ), zeatin (cZ), trans-zeatin riboside (tzR), and zeatin riboside (czR). Most hormones were not induced by DSF except iP and iPR (downregulation) (**Figures 5H, I**). However, when *Xcc* infected the seedlings, the level of JA, an important defensive hormone, and its amino acid derivative JA-Ile increased, sharply triggered by DSF pretreatment (**Figures 5A, B**). ABA and zeatin biosynthesis hormones tZ and cZ were also promoted by DSF in *Xcc* -challenged seedlings, while IAA and iPR were decreased (**Figures 5D–G, I**). Another important defensive hormone SA was elevated by *Xcc* invasion but was not affected by DSF pretreatment (**Supplementary Figure 3A**). The content of *cis*-OPDA, the precursor of JA (Taki et al., 2005), was also insensitive to DSF treatment whether or not in the *Xcc* challenge condition (**Figure 5C**). Other phytohormones including ACC, tZR, and cZR were not influenced by DSF and *Xcc* infection (**Supplementary Figures 3B–D**).

### DSF primes JA accumulation by eliciting JA synthesis gene expression

**Figure 6:**
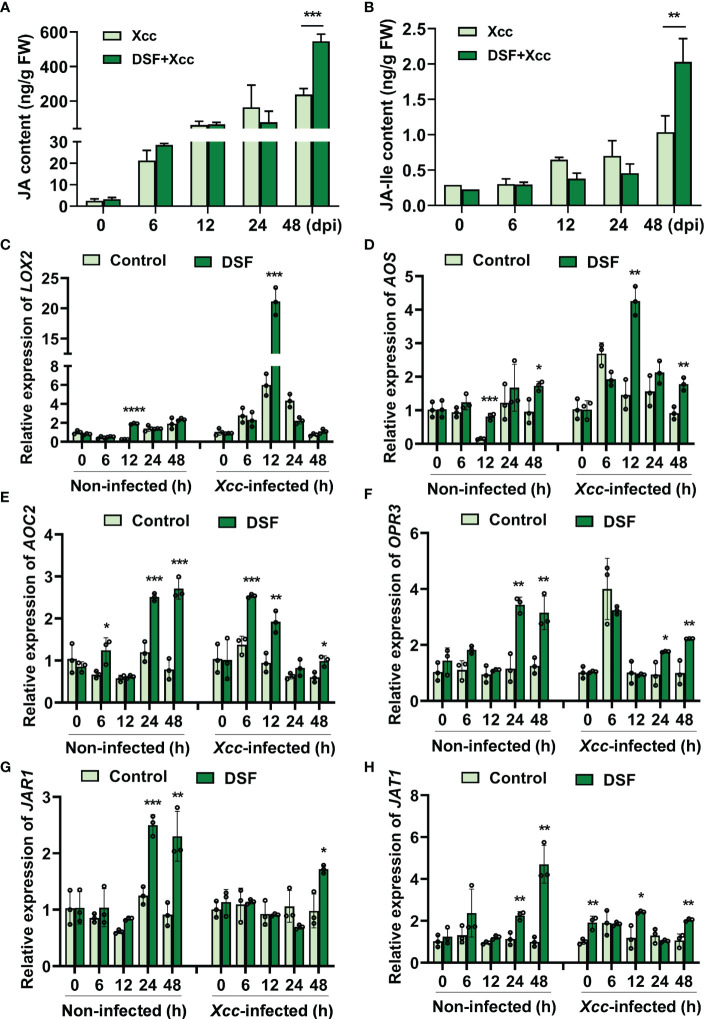
Effects of priming by DSF and pathogen challenge on JA accumulation. The seedlings were pretreated with 2 µM of DSF for 48 h prior to inoculation with 10^8^ CFU/ml of *Xcc*. Samples were harvested at indicated time points. **(A)** The content of JA. **(B)** The content of JA-Ile. **(C–F)** Expression of JA synthesis genes *LOX2*, *AOS*, *AOC2*, and *OPR3*. **(G)** The expression of JA-Ile synthesis gene *JAR1*. **(H)** The expression of JA transporter gene *JAT1*. Values are means ± SD of three independent experiments. Asterisks indicate statistically significant differences (ANOVA test, *p < 0.05, **p < 0.01, ***p < 0.001, ****p < 0.0001). DSF, diffusible signal factor; CFU, colony-forming unit.

To further verify the effects of DSF on JA accumulation, the content of JA was determined at different times after *Xcc* inoculation in *Arabidopsis*. *Xcc* triggered the level of JA to rise rapidly when inoculation time was ≥ 6 hpi. DSF pretreatment also elicited JA concentration to rise simultaneously but had no significant difference when compared to the *Xcc* challenge only. At 48 hpi, the priming effect of DSF on promoting JA accumulation was remarkable (**Figure 6A**). JA-Ile is the most bioactive form of JAs (Fonseca et al., 2009). The effect of DSF on JA-Ile was similar to that of JA (**Figure 6B**).

In *Arabidopsis*, three enzymes in chloroplast are required including lipoxygenase (LOX), allene oxide synthase (AOS), and allene oxide cyclase (AOC) in JA biosynthesis pathways (Ruan et al., 2019). The gene expression of *LOX2*, *AOS*, and *AOC2* was detected by qPCR in seedlings pretreated by DSF prior to *Xcc* infection. DSF induced *LOX2* upregulation dramatically at 12 hpi (**Figure 6C**). At 12 and 48 hpi, the expression of *AOS* and *AOC2* was also increased by DSF (**Figures 6D, E**). OPR3 catalyzes OPDA and subsequently with three rounds of β-oxidation to yield JA [(+)-7-iso-JA] (Liu and Timko, 2021). *OPR3* was induced by DSF at 24 and 48 h after DSF treatment and subsequently with the *Xcc* challenge (**Figure 6F**). Jasmonoyl-isoleucine synthetase (JAR1) catalyzes the conjugation of JA to the amino acid isoleucine to form JA-Ile (Fonseca et al., 2009). The expression of *JAR1* was increased significantly by DSF at 24 and 48 h prior to *Xcc* infection and at 48 h after *Xcc* infection (**Figure 6G**). The ABC transporter JAT1 with JA transport ability mediates the bioactive JA-Ile across the inner membrane of the nuclear to activate JA responses (Ruan et al., 2019). The expression of *JAT1* was increased significantly by DSF at 24 and 48 h prior to *Xcc* infection and at 12 and 48 h after *Xcc* infection (**Figure 6H**). These results indicated that DSF promotes JA and JA-Ile accumulation in plant cells by eliciting the expression of JA synthesis and transport genes.

### DSF modulated the expression of JA signaling genes

**Figure 7:**
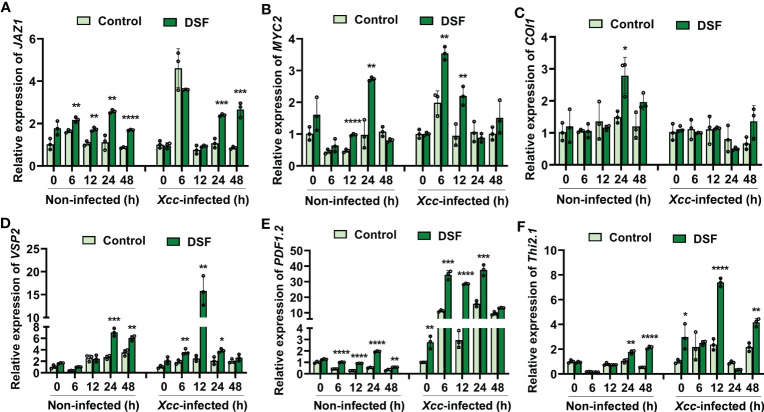
Expression of JA signaling genes primed by DSF with *Xcc* challenge in *Arabidopsis*. The seedlings were pretreated with 2 µM of DSF for 48 h prior to inoculation with 10^8^ CFU/ml of *Xcc*. Samples were harvested at indicated time points for qPCR. **(A–C)** Expression of JA signaling transcriptional regulator genes *JAZ1*, *MYC2*, and *COI1*. **(D–F)** Expression of JA signaling downstream cascade genes *VSP2*, *PDF1.2*, and *Thi2.1*. Values are means ± SD of three independent experiments. Asterisks indicate statistically significant differences (ANOVA test, *p < 0.05, **p < 0.01, ***p < 0.001, ****p < 0.0001). DSF, diffusible signal factor; CFU, colony-forming unit.

Coronatine insensitive 1 (COI1) and jasmonate ZIM-domain protein (JAZ) are coreceptors of JA signaling (Sheard et al., 2010). The basic helix-loop-helix (bHLH) transcription factor MYC2 is a well-known regulatory protein of JA signaling (Ruan et al., 2019). DSF pretreatment elicited the expression level of *JAZ1* to rise at 24 and 48 hpi (**Figure 7A**). The transcriptional level of *MYC2* was promoted by DSF at 6 and 12 hpi (**Figure 7B**). The expression of *COI1* was not influenced by DSF pretreatment following *Xcc* infection (**Figure 7C**). The expression of JA-responsive marker genes *VSP2*, *PDF1.2*, and *Thi2.1* was detected in seedlings pretreated by DSF prior to *Xcc* infection. VSP2 has acid phosphatase activity dependent on the presence of a divalent cation. *PDF1.2* encodes ethylene- and JA-responsive plant defense. *Thi2.1* encodes a thionin, which is a cysteine-rich protein having antimicrobial properties (Liu et al., 2022). The expression levels of *VSP2*, *PDF1.2*, and *Thi2.1* were induced by DSF treatment slightly and increased dramatically at 12 h after *Xcc* inoculation in seedlings primed with DSF (**Figures 7D–F**). These results indicated that DSF primed plant resistance against *Xcc* by activating the expression of JA signaling genes.

### The DSF priming effect is absent in JA signaling mutants *coi1-1* and *jar1-1*

**Figure 8:**
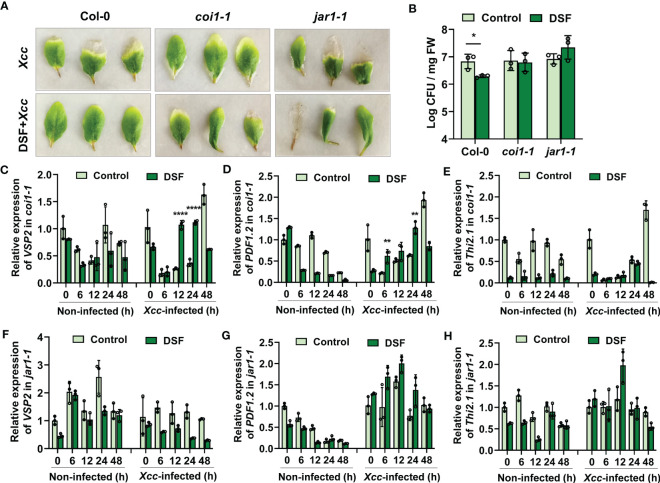
Desensitization of priming effects elicited by DSF and *Xcc* challenge in JA signaling mutants *coi1-1* and *jar1-1*. The seedlings were pretreated with 2 µM of DSF for 48 h prior to inoculation with 10^8^ CFU/ml of *Xcc*. **(A)** Symptoms of *Xcc* infection on *Arabidopsis* mutants *coi1-1* and *jar1-1* pretreated with 2 µM of DSF. The disease symptoms were recorded at 72 h after inoculation. **(B)** Proliferation of *Xcc* in the leaves of wild-type *Arabidopsis* Col-0 and mutants *coi1-1* and *jar1-1* at 48 hpi. **(C–E)** Expression of JA- responsive genes *VSP2*, *PDF1.2*, and *Thi2.1* in mutant *coi1-1*. **(F–H)** Expression of JA- responsive genes *VSP2*, *PDF1.2*, and *Thi2.1* in mutant *jar1-1*. Values are means ± SD of three independent experiments. Asterisks indicate statistically significant differences (ANOVA test, *p < 0.05, **p < 0.01, ****p < 0.0001). DSF, diffusible signal factor; CFU, colony-forming unit; hpi, post-inoculation.

To further investigate the role of JA signaling in DSF- primed resistance to *Xcc*, two functional defective mutants *coi1-1* and *jar1-1* were employed for comparison with wild-type Col-0. COI1 is the JA signaling receptor, and *coi1-1* is defective in JA perception (Yang et al., 2019). JAR1 plays an important role in transforming JA into bioactive JA-Ile, and *jar1-1* is defective in the synthesis of JA-Ile (Chini et al., 2007). JA-Ile binds with the COI1-JAZ complex to activate JA signaling (Ruan et al., 2019). DSF pretreatment could relieve the *Xcc* disease symptoms in detached leaves of wild-type *Arabidopsis* Col-0, while the priming- resistant effect of DSF was not observed in mutants *coi1-1* and *jar1-1* (**Figure 8A**). Consistently, there was no significant difference in the *Xcc* proliferation levels between DSF- pretreated seedlings and un-pretreated ones in *coi1-1* and *jar1-1*, while a reduction of bacterial proliferation was observed in wild-type seedlings pretreated by DSF (**Figure 8B**). Unlike wild-type Col-0, the expression of JA- responsive genes *VSP2* and *PDF1.2* only increased slightly, while the expression of *Thi2.1* was not increased by DSF in mutant *coi1-1* (**Figures 8C–E**). The expression levels of these three JA- responsive genes were also not increased by DSF pretreatment prior to *Xcc* infection in mutant *jar1-1* (**Figures 8F–H**). These results indicated that the synthesis and perception of the bioactive JA-Ile were required for the DSF priming process.

### DSF primes resistance against *Xcc* in cabbage (*B. oleracea* L.)

**Figure 9:**
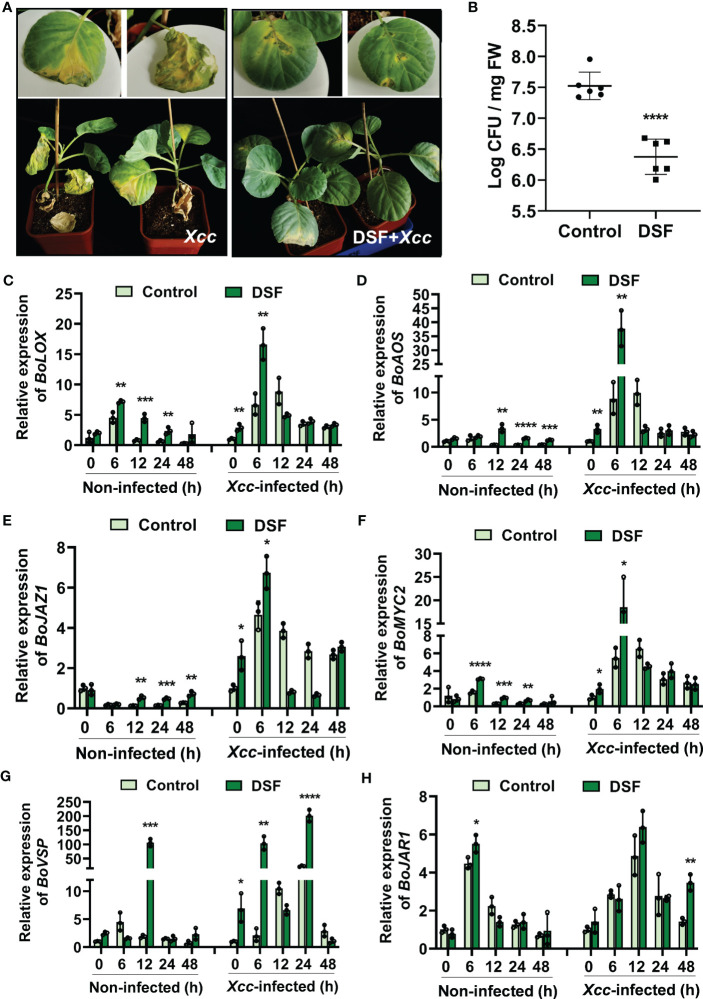
Enhanced resistance of DSF-treated cabbage against *Xcc*. The seedlings were pretreated with 2 µM of DSF for 48 h prior to inoculation with 10^8^ CFU/ml of *Xcc*. **(A)** Symptoms of *Xcc* infection on cabbage pretreated with 2 µM of DSF in pot-cultured seedlings. The disease symptoms were recorded 21 days after inoculation. **(B)** Proliferation of *Xcc* in the leaves of cabbage at 48 hpi. **(C, D)** Expression of JA synthesis genes *BoLOX* and *BoAOS*. **(E, F)** Expression of JA regulator genes *BoJAZ1* and *BoMYC2*. **(G)** The expression of JA- responsive gene *BoVSP*. **(H)** The expression of JA-Ile synthesis gene *BoJAR1*. Values are means ± SD of three or six independent experiments. Asterisks indicate statistically significant differences (ANOVA test, *p < 0.05, **p < 0.01, ***p < 0.001, ****p < 0.0001). DSF, diffusible signal factor; CFU, colony-forming unit; hpi, post-inoculation.

To explore the priming effects of DSF against black rot in *B. oleracea*, detached leaves and soil-cultured pot seedlings were pretreated with 2 µM of DSF for 48 h and then inoculated with 1 × 10^8^ CFU/ml of pathogen *Xcc* by suspension inoculation method and leaf cutting inoculation method. The disease symptoms were observed after 9 days in detached leaves and 21 days in pot seedlings. The leaves infected by *Xcc* showed a typical black rot symptom with a filemot necrotic lesion, while DSF- pretreated leaves exhibited no significant *Xcc* symptoms (**Figure 9A** and **Supplementary Figure 4**). *Xcc* proliferation in *B. oleracea* leaves was significantly inhibited by DSF (**Figure 9B**). It indicated that DSF plays an important role in priming *B. oleracea* resistance against pathogen *Xcc*.

In view of DSF priming plant resistance dependent on the JA signaling pathway in *Arabidopsis*, JA signaling orthologues were detected in *B. oleracea*. JA biosynthesis genes *BoLOX* and *BoAOS* were strongly induced by DSF, especially when they underwent pathogen *Xcc* challenge at 6 hpi (**Figures 9C, D**). The expression of JA signaling transcriptional regulator genes *BoMYC2* and *BoJAZ1* were also increased by DSF whether or not under *Xcc* infection condition (**Figures 9E, F**). *BoVSP*, one of the JA- responsive genes, was all upregulated drastically by DSF with fold change of up to 100 when DSF was treated at 12 h and DSF-pretreated prior to *Xcc* invasion at 6 and 24 hpi (**Figure 9G**). *BoJAR1*, which functions on JA-Ile synthesis, was induced significantly by DSF under the *Xcc* challenge (**Figure 9H**). These results suggested that JA signaling may be involved in DSF- primed *B. oleracea* resistance against *Xcc*.

## Discussion

Plants and bacteria have co-existed for millions of years, and complex networks consisting of different signaling molecules have evolved. QS is one of the best-studied cell–cell communication modes (He et al., 2023). Accumulating evidence suggests that QS signaling molecules such as DSF mediate not only intraspecies communication and interspecies communication within microbes but also inter-kingdom communication between DSF-producing bacteria and their eukaryotic hosts (Kakkar et al., 2015; Tran et al., 2020; Ma et al., 2021; Zhai et al., 2022; He et al., 2023). Kakkar et al. (2015) reported that DSF signal induced innate immunity against pathogen infection in a dose-dependent manner in *N. benthamiana*, *Arabidopsis*, and rice. High dose of DSF (≥100 μM) induced HR-like symptoms, callose deposition, programmed cell death, H_2_O_2_ accumulation, and *PR-1* gene expression, while a low dose of DSF (≤10 μM) did not induce a significant amount of callose deposition (Kakkar et al., 2015). Tran et al. (2020) found that 25 μM of DSF, mimicking early-infection concentrations, hijacked *Arabidopsis* sterol biosynthesis, thereby suppressing PTI responses, including callose deposition, ROS production, and stomatal closure. However, pre-infiltration with 10 μM of DSF and subsequent challenge with flg22 caused a substantial amount of callose deposition, which suggested that the application of a lower concentration of DSF may prime plants and influence their subsequent defense response (Kakkar et al., 2015). In this study, we demonstrated that DSF could prime plant immunity effectively against *Xcc* in both *A. thaliana* and *B. oleracea* with a low concentration (1–5 μM) (**Figures 1**, **9**). It was found that 2 μM of DSF pretreatment decreased the disease symptoms and *Xcc* proliferation on plant leaves and upregulated the transcriptional level of PTI marker genes *WRKY22* and *FRK1* (**Figures 1**, **9**). These results indicated that DSF plays an important role in priming plant resistance against the *Xcc* pathogen and that the concentration of 2 μM was the optimized dose of DSF to prime plant immunity.

ROS production is critical for plant development, growth, and response to abiotic and biotic stress (Wang et al., 2018). Plant NADPH oxidases, the respiratory burst oxidase homologs (RBOHs), which generate superoxide anions ( ${\text{O}}_{2}^{\;-}$ ) in the apoplast that rapidly dismutated to H_2_O_2_, are a major source of ROS during plant–microbe interactions (Otulak-Kozieł et al., 2020). RBOHD and RBOHF are pleiotropic and function together, contributing to stress signaling, especially pathogen response differentially modulating the immune response (Morales et al., 2016). In this study, DSF treatment elicited ROS burst in the *Arabidopsis* root, and CAT application proved that the cellular level of H_2_O_2_ was elevated mainly by DSF (**Figure 2**). ROS burst, H_2_O_2_ accumulation, antioxidase POD activation, and *RBOHD/F* upregulation were observed after DSF treatment followed by *Xcc* inoculation in *Arabidopsis* leaves (**Figure 3**). These pieces of evidence indicated that RBOH-mediated ROS production plays an important role in DSF-primed plant resistance.

JA and its metabolic derivatives, such as JA-Ile and methyl jasmonate (MeJA), collectively known as jasmonates (JAs), are a class of lipid-derived, natural, and widely distributed phytohormones in higher plants (Liu and Timko, 2021). JA and its derivatives are key signaling compounds closely related to plant defense and resistance to microbial pathogens, herbivorous insects, wounding, light, drought, salt, ozone stress, and low temperature (Rao et al., 2000; Balbi and Devoto, 2008; Glauser et al., 2008; Mewis et al., 2012; Ellouzi et al., 2013; Zhao et al., 2013; Ruan et al., 2019; Wang et al., 2019). In *Arabidopsis*, there are three pathways for the synthesis of JAs (Chini et al., 2018; Liu and Timko, 2021). The octadecane pathway starting from α-linolenic acid (α-LeA, 18:3) was the main pathway. Through a sequential series of reactions catalyzed by LOX, AOS, and AOC, α-LeA is converted to OPDA. Then, OPDA is transported from chloroplast to peroxisome, where it is reduced by OPR3 and subsequently shortened by three rounds of β-oxidation, finally yielding JA [(+)-7-iso-JA] (Liu and Timko, 2021). In the cytoplasm, JA is metabolized into different structures by various chemical reactions, such as MeJA, JA-Ile, *cis*-jasmone (CJ), and 12-hydroxyjasmonic acid (12-OH-JA), and *JAR1*, *CYP94B3*, and *CYP94C1* are involved in this process (Ruan et al., 2019). JAT1 mediated the bioactive JA-Ile transport quickly into the nucleus when the plant is under stress (Ruan et al., 2019). COI1-JAZ complex is a high-affinity receptor for the bioactive JA-Ile (Sheard et al., 2010). COI1 associates with the SKP1 protein and Cullin protein to form the SCF-type E3 ubiquitin ligase SCF^COI1^, which targets JAZ for degradation by ubiquitination (Zhai et al., 2015). Subsequently, JAZ proteins are degraded after being transferred to the 26S proteasome, and simultaneously, transcription factors such as MYC2 are released to activate the expression of downstream genes (Ruan et al., 2019). In this study, transcriptome combined with metabolome analysis showed that plant hormone JA signaling was involved in DSF-primed resistance to *Xcc* in *Arabidopsis* (**Figures 4**, **5**). Biosynthesis, metabolism, and signaling genes of JA including *LOX*, *JAZ*, *VSP2*, *CYP94B3*, and *CYP94C1* were induced by DSF priming following *Xcc* invasion (**Figures 4**, **6**, **7**). *LOX2*, *AOS*, *AOC2*, *JAR1*, *JAT1*, *JAZ1*, and *MYC2* were all induced in the DSF priming process in *Arabidopsis* and cabbage (**Figures 6**, **7**, **9**). Elevated JA and JA-Ile hormone levels and desensitizing of JA signaling mutants *coi1-1* and *jar1-1* (**Figures 6**, **8**) confirmed that DSF-primed resistance against *Xcc* was dependent on the JA pathway.

DSF-producing phytopathogens or plant-associated bacteria use DSF signals to affect host cell biology through multilayered regulatory networks (He et al., 2023). The regulatory mechanism of DSF during the *Xanthomonas*–plant interactions is far from clear. The role of JA and JA derivatives in DSF-mediated *Xanthomonas*–plant interactions needs further study. JA signaling crosstalk with other phytohormones such as SA, auxin, ABA, and zeatin in DSF priming plant immunity needs more investigation. Moreover, the role of calcium signaling, G protein signaling, and MAPK signaling in DSF-mediated *Xanthomonas*–plant interactions is also the direction of further research. Understanding the mechanisms of QS signal-mediated communication has important implications for appreciating host–pathogen interactions and providing new strategies for the control of plant disease.

## Data availability statement

The datasets presented in this study can be found in online repositories. The names of the repository/repositories and accession number(s) can be found in the article/**Supplementary Material**.

## Author contributions

QZ and SS drafted the manuscript. QZ, FL, and CS performed the plant pathology analysis, molecular analysis, and genetic assay, respectively. TZ, ZH, LM, XZ, and ZJ helped with the acquisition, analysis, and interpretation of data for the work. SS was responsible for the design of the work. All authors gave approval to the final version.

## Supplementary material

Supplementary Table 3 DSF_vs_CK.DEG_GO_enriched_result (padjust 0.05).

Supplementary Table 4 DSF-Xcc_vs_CK-Xcc.DEG_GO_enriched_result (padjust 0.05).

Supplementary Table 5 DSF_vs_CK.DEG_KEGG_enriched_result.

Supplementary Table 6 DSF-X_vs_CK-X.DEG_KEGG_enriched_result.
