# Supplementary material for: Diffusible signal factor primes plant immunity against Xanthomonas campestris pv. campestris (Xcc) via JA signaling in Arabidopsis and Brassica oleracea
Source: Front Cell Infect Microbiol. 2023 Jun 19;13:1203582. doi: 10.3389/fcimb.2023.1203582 (PMC10315614; doi:10.3389/fcimb.2023.1203582)
Supplement: Supplementary file 1 [file DataSheet_1.pdf]

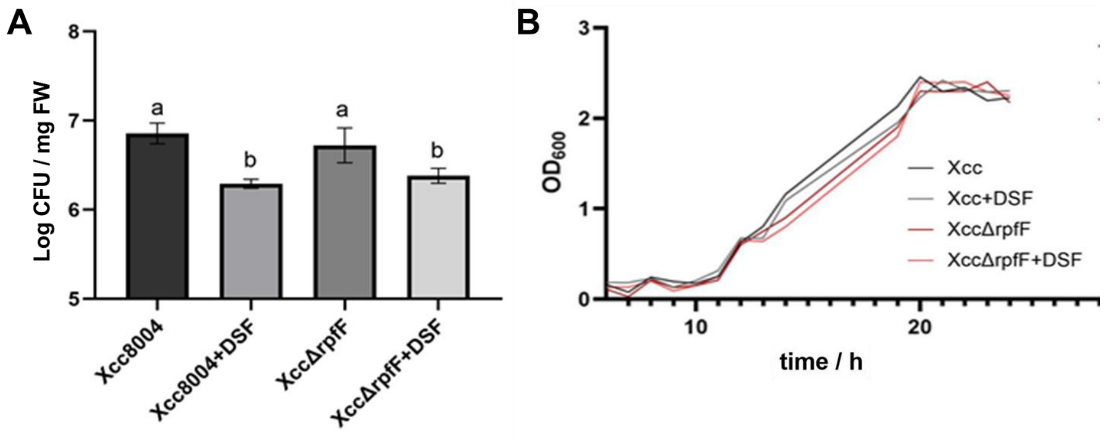

Supplementary figure 1: The influence of endogenous DSF on DSF priming effect in Arabidopsis. (A) Proliferation of *Xcc* in the leaves of wild-type Arabidopsis Col-0. The seedlings were pretreated with 2 μM DSF for 48 h prior to inoculation with 10^8 CFU/mL *Xcc8004* (wild-type strain) or *Xcc*Δ*rpfF* (mutant strain that lack of DSF synthesis). (B) The growth curve of *Xcc8004* and *Xcc*Δ*rpfF* with 2 μM exogenous DSF application or not. Values are means ± SD of three independent experiments. Different letters indicate statistically significant differences (ANOVA test, $P < 0.05$).
