# Supplementary material for: Diffusible signal factor primes plant immunity against Xanthomonas campestris pv. campestris (Xcc) via JA signaling in Arabidopsis and Brassica oleracea
Source: Front Cell Infect Microbiol. 2023 Jun 19;13:1203582. doi: 10.3389/fcimb.2023.1203582 (PMC10315614; doi:10.3389/fcimb.2023.1203582)
Supplement: Supplementary file 2 [file DataSheet_2.pdf]

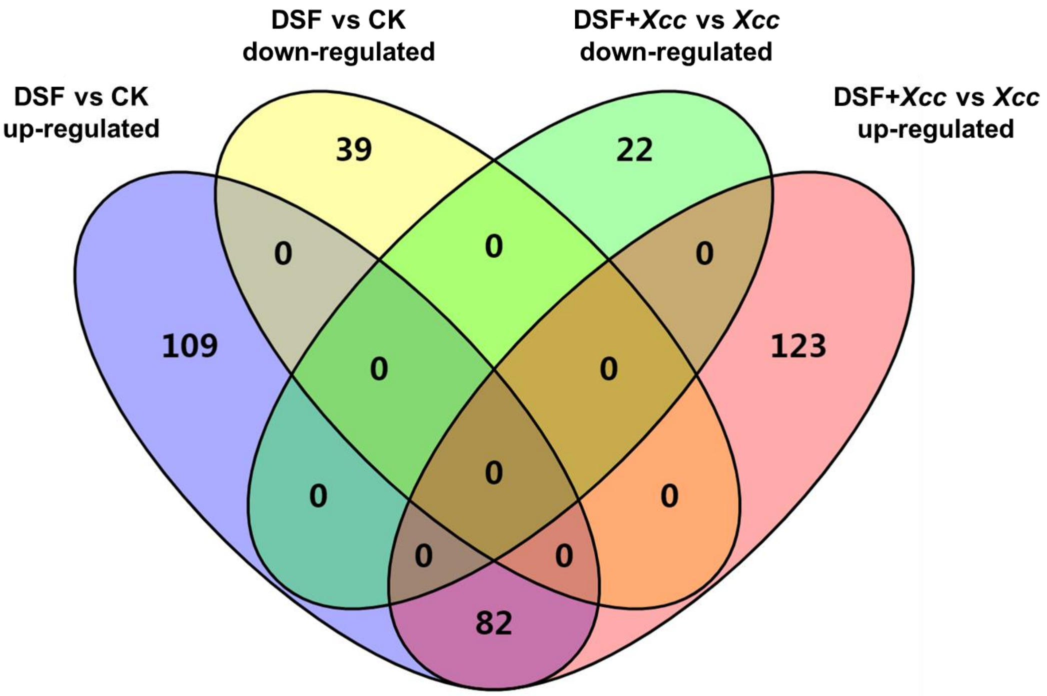

Supplementary figure 2: Differential gene expression in response to DSF and following with *Xcc* infection. The seedlings were pretreated by DSF for 48 h following *Xcc* infection for another 48 h. DSF untreated seedlings with synchronous growth were as blank control and *Xcc* inoculated seedlings without DSF pre-treatment were as pathogen control. Total 230 differentially expressed genes (DEGs) including 191 upregulated genes and 39 downregulated genes were identified in DSF treated seedlings compared to untreated control. And there were total 227 DEGs including 205 genes upregulation and 22 genes downregulation in DSF pretreated seedlings prior to *Xcc* challenge compared to *Xcc* inoculated seedlings without DSF pre-treatment. 82 upregulated DEGs were found both in DSF-treated seedlings and in DSF-pretreated seedlings with *Xcc* infection.
