# Supplementary material for: Diffusible signal factor primes plant immunity against Xanthomonas campestris pv. campestris (Xcc) via JA signaling in Arabidopsis and Brassica oleracea
Source: Front Cell Infect Microbiol. 2023 Jun 19;13:1203582. doi: 10.3389/fcimb.2023.1203582 (PMC10315614; doi:10.3389/fcimb.2023.1203582)
Supplement: Supplementary file 3 [file DataSheet_3.pdf]

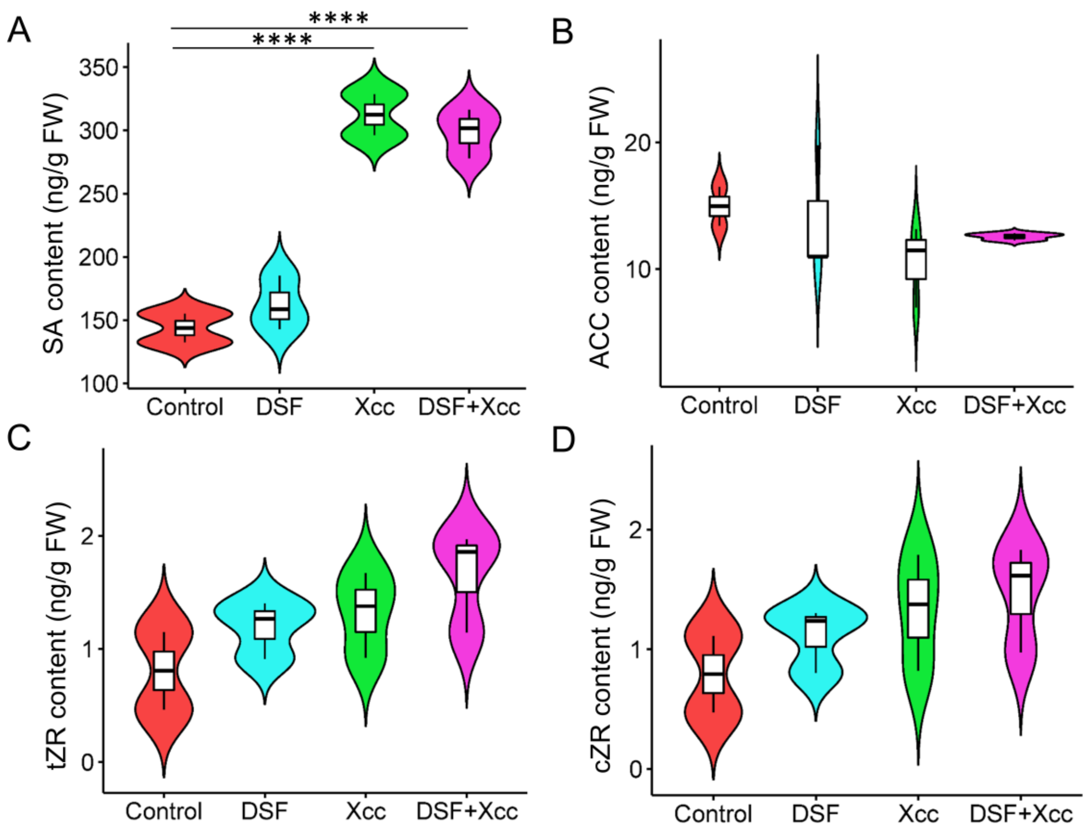

Supplementary figure 3: The level of phytohormones in response to DSF and following with *Xcc* infection. The seedlings were pretreated with 2 μM DSF for 48 h prior to inoculation with 10^8 CFU/mL *Xcc*. Phytohormones were extracted to test the level changes between DSF treatment and untreated control (48 h), or between DSF pretreatment and un-pretreatment prior to Xcc infection (48 hpi). (A) SA, (B) ACC, (C) tZR, (D) cZR. Values are means of three duplicate sample data. Astersks indicate a statistically significant differences (ANOVA test, ****P<0.0001).
