# Supplementary material for: Diffusible signal factor primes plant immunity against Xanthomonas campestris pv. campestris (Xcc) via JA signaling in Arabidopsis and Brassica oleracea
Source: Front Cell Infect Microbiol. 2023 Jun 19;13:1203582. doi: 10.3389/fcimb.2023.1203582 (PMC10315614; doi:10.3389/fcimb.2023.1203582)
Supplement: Supplementary file 4 [file DataSheet_4.pdf]

Supplementary Table 1. Primer information of genes investigated in qRT-PCR

Gene	Forward primer (5’→3’)	Reverse primer (5’→3’)
AOC2	TCGTAAGCGTAATGTGTCCCGTCCCT	CGAGCCTACTAAGCCAAACTTTCCAA
AOS	TCCGACGGTGGGGAATAAACAATG	AACAGATGGACTACACAGGTGCGAAC
LOX2	CAGAGCAACGCTACGGGGGAGAG	AGAACTGGGGCATCAAACTGGAGAAT
LOX3	CAGGAGATGCGGAGATTGTTG	GGCTCAGAACTCGGAACCA
LOX4	TTTTTAGGGGGATGGCTGT	AGAGACCGTCGTTGGCGTA
VSP2	AGCATCTCATACTCAAGCCAAACG	AGTATCCTCAACCAAATCAGCCCA
Thi2.1	GTTGGGTAAACGCCATTCTC	CCCAGGTGGGACTACATAGC
MYC2	TTGATGAGGAGGTGACGGATAC	CCAAACACTCCTCCTTGCTTAG
PDF1.2	CATGGCTAAGTTTGCTTCCA	GTTGCATGATCCATGTTTGG
WRKY22	CAAAAGGTTGTTTAGCCCGTA	ATGATTATGCTCCGCCGTGTA
FRK1	AGATGGCGGACTTCGGGTTA	CACTCTTTTCGTTCATTTGGCG
RBOHD	CAATAGTGTTGCTGGCGGC	GGCGTTCTTGATGCGTGAG
RBOHF	TTTGTCCAATGTCCTGCGG	TCGTCGGCTCTGAGAAGTCC
OPR3	ATTATGGCATGTTGGACGTG	AACAAAACTCGCCACCTGTT
CYP94B3	TGGCTTACACGAAGGCTTGTC	AGTCCCACGAAACTGGAGGAT
CYP94C1	GGCCCGGATTACGAAGAGTTT	GGCCGGAACTTACCTTCGTT
JAZ1	CTTTTATGCCGGTTCAGTTTGTGTT	GCTTTTGTGGCTCCGAGTCCGTTTG
JAZ10	CAACATCTCCTTACTATCCGACAT	ACGATTTAGCAACGACGAAGAA
JAZ13	GCCTCTACGCTTCAATCTTGTC	CGGCACTAACTCTAACGCTGT
COI1	CTTCTACATGACGGAGTTTGCC	CACCGACCTTCACAGATACCAG
JAR1	AACGCTACTGACCCTGAAGAAGC	GGTGAAGTGTCACCATCAACCA
JAT1	TGGTTCTTCACTTGACGGAGAT	AAGAGTCTTGGTCTTGGAGA
ACTIN2/8	CCAGAAGGATGCATATGTTGGTGA	GAGGAGCCTCGGTAAGAAGA
BoAOS	AGGCTGGAGAAATGCTTTATGG	GCAACAACCTCTCTCCTTCCTC
BoLOX	GCGGCTAACGATACCAGACT	CTATGTTCCTCACTTCACTCCAC
BoVSP	CCCGAATGCGTTCAATAAGTGG	AGTAAGACAGGCTCAATCCCGA
BoMYC2	TCGTAAGAAGGTGTTGCGTGAG	GACCCATAAACCCAAACTGCG

BoJAZ1	ACAACATCGCTCCTACCCCA	ACCCTATCCTTTCTCTTCGCCA
BoJAR1	GAGACAACACGACAGACCCA	CTGAAGGCAAAAGCAGTACGAA
BoACT1	CACTGTTCCAATCTACGAGGGT	CACAGCGACAAAGGAGAGC
