# Supplementary material for: Diffusible signal factor primes plant immunity against Xanthomonas campestris pv. campestris (Xcc) via JA signaling in Arabidopsis and Brassica oleracea
Source: Front Cell Infect Microbiol. 2023 Jun 19;13:1203582. doi: 10.3389/fcimb.2023.1203582 (PMC10315614; doi:10.3389/fcimb.2023.1203582)
Supplement: Supplementary file 5 [file DataSheet_5.pdf]

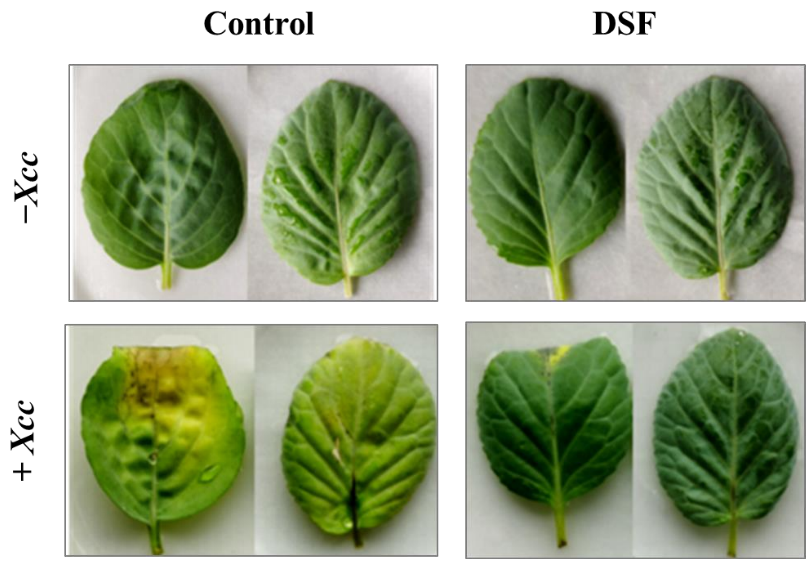

Supplementary figure 4: Symptoms of *Xcc* infection on *Brassica oleracea* detached leaves. *Brassica oleracea* that soil-cultured in four leaves and one shoot period were used for treatments. Detached leaves were soaked in sterile dH_2O (Control) or 2μM DSF for 48 h, then inoculated with 1×10^8 CFU/ml *Xcc* and cultured at 28℃. The disease symptoms were recorded at 9 d after inoculation.
