# Supplementary material for: Diffusible signal factor primes plant immunity against Xanthomonas campestris pv. campestris (Xcc) via JA signaling in Arabidopsis and Brassica oleracea
Source: Front Cell Infect Microbiol. 2023 Jun 19;13:1203582. doi: 10.3389/fcimb.2023.1203582 (PMC10315614; doi:10.3389/fcimb.2023.1203582)
Supplement: Supplementary file 6 [file DataSheet_6.pdf]

Supplementary Table 2. Verification of DEGs induced by DSF in RNAseq by qPCR.

Genes	Gene ID	RNAseq log2Fold	qPCR ratio	Discription
JAZ10	AT5G13220	3.6047	6.411	JA signaling
LOX4	AT1G72520	2.9375	8.294	JA signaling
CML40	AT3G01830	2.3525	3.927	Ca^{2+} signaling
MAPKKK21	AT4G36950	3.3132	3.844	MAPK signaling
CML37	AT5G42380	1.7972	6.292	Ca^{2+} signaling
CNGC19	AT3G17690	1.9333	13.706	Ca^{2+} signaling
PEPR2	AT1G17750	2.0124	8.006	Leucine-rich repeat receptor-like protein kinase
PEPR1	AT1G73080	1.6513	6.85	Defense related
ERF016	AT5G21960	2.3476	7.661	Ethylene signaling
PBS3	AT5G13320	-1.0566	0.073	Auxin signaling
