# Supplementary material for: Diffusible signal factor primes plant immunity against Xanthomonas campestris pv. campestris (Xcc) via JA signaling in Arabidopsis and Brassica oleracea
Source: Front Cell Infect Microbiol. 2023 Jun 19;13:1203582. doi: 10.3389/fcimb.2023.1203582 (PMC10315614; doi:10.3389/fcimb.2023.1203582)
Supplement: Supplementary file 7 [file DataSheet_7.pdf]

Supplementary Table 7: The ortholog genes in *Brassica oleracea* correspond to in Arabidopsis.

In *Brassica oleracea*		In Arabidopsis	
Ortholog genes	GenBank ID	Genes	Gene ID
BoAOS	JQ771611.1	*AtAOS*	AT5G42650
BoLOX	EF123056	*AtLOX2*	AT3G45140
BoVSP	EU921650.1	*AtVSP2*	AT5G24770
BoMYC2	XM_013731272	*AtMYC2*	AT1G32640
BoJAZ1	XM_013728482	*AtJAZ1*	AT1G19180
BoJAR1	XM_013781405	*AtJAR1*	AT2G46370
